# The gastrointestinal tract of farmed mink (*Neovison vison*) maintains a diverse mucosa‐associated microbiota following a 3‐day fasting period

**DOI:** 10.1002/mbo3.434

**Published:** 2017-01-16

**Authors:** Martin I. Bahl, Anne S. Hammer, Tove Clausen, Anabelle Jakobsen, Søren Skov, Lars Andresen

**Affiliations:** ^1^ Division of DietDisease prevention and ToxicologyNational Food InstituteTechnical University of DenmarkSøborgDenmark; ^2^ Department of Veterinary Disease BiologyFaculty of Health and Medical SciencesUniversity of CopenhagenFrederiksberg CDenmark; ^3^ Danish Fur Breeders Research CentreHolstebroDenmark

**Keywords:** gut, microbiota, microflora, mink, *Neovison vison*

## Abstract

Although it is well documented that the gut microbiota plays an important role in health and disease in mammalian species, this area has been poorly studied among carnivorous animals, especially within the mustelidae family. The gastrointestinal tract of carnivores is characterized by its short length and fast transit time, as compared to omnivores and herbivores, which is due to the low level of inherent fermentation. Mink represents an example of this, which have a GI tract only four times the length of the body and a transit time of approximately 4–5 hr. In this study, we used high‐throughput 16S rRNA gene sequencing to explore the resident gut microbiota of the mink in terms of intra‐and interindividual diversity. We report, for the first time, that the mucosa‐associated bacterial community within the colon is diverse and dissimilar from the community found in the feed. We found large interindividual differences in bacterial composition between individual animals being dominated generally by the phylum Firmicutes, but in some cases also Proteobacteria or Fusobacteria. The bacterial load and community structure within the mucus was not severely impacted by 3 days of fasting, which implies that a resident and stable microbiota is hosted by these animals.

## 1 INTRODUCTION

The gut microbial community composition of carnivores in general appears distinct from that of omnivores and herbivores (Ley et al., 2008) and is vastly dominated by species belonging to the Firmicutes phylum, and comparatively few Bacteroidetes as demonstrated by 16S rRNA gene sequencing of fecal samples from, for example, cats and dogs (Garcia‐Mazcorro et al., 2012; Handl et al., 2011). A recent culture‐based study of the anaerobic and microaerophilic bacteria in fecal samples from ferrets (*Mustela putorius furo*) found *Clostridium acetobutylicum* and *Helicobacter* spp., respectively, to be the most frequently isolated (Nizza et al., 2014). Looking more specifically at the mink microbiota (*Neovison vison*), it has been shown by culturing that the highest bacterial load was found in the colon with up to 10^8^ CFU/g, which is approximately 2–4 orders of magnitude lower than that found in many other mammals, which could be due to the short intestinal tract and fast transit time in the gut (Williams, Elnif, & Buddington, 1998). The intestinal tract of the mink is only four times the body length and the transit time 4–5 hr (Hansen, 1978; Szymeczko & Skrede, 1990). In a more recent study, even shorter transit times were recorded for farmed mink and appeared to vary between males and females (Gugołek et al., 2013). There have been few investigations of the mink gut microbiota and they have mostly applied conventional culture‐based methods (Vulfson et al., 2001, 2003; Williams et al., 1998). In these studies, bacteria were cultured from most, but not all samples and bacterial composition and counts were shown to vary significantly through the growth season. Bacterial findings were also affected by the age and feed of the animals (Vulfson et al., 2001, 2003).

Depriving animals of feed for a period of time has previously been shown to affect the microbiota composition in a number of phylogenetically diverse species including fish, rodents, and dogs (Kasiraj et al., 2016; Kohl et al., 2014; Sonoyama et al., 2009) which reflects a microbial response to the changed nutritional environment. Reported effects on the microbiota following feed deprivation include increase of the mucin‐degrading bacteria *Akkermansia muciniphila* (Sonoyama et al., 2009) and *Bacteroidetes* (Kasiraj et al., 2016), which may both have a competitive advantage in the low nutrient environment, due to utilization of host‐derived mucus glycans (Derrien et al., 2004; Sonnenburg et al., 2005). No universal perturbation effect has been found across all species, which in part is caused by differences in baseline microbiota composition. Additionally, reduced concentrations of short‐chain fatty acids (microbial fermentation products) in the cecum during fasting have been reported (Sonoyama et al., 2009). Changes induced by fasting in canine jejunal microbiota composition have been shown to subside once the animals return to normal feeding (Kasiraj et al., 2016). This indicates an overall resilience to this form of perturbation, similar to what is often observed following antibiotic treatment (Dethlefsen et al., 2008). Here, we investigate the structure and resilience of the mink mucosa‐associated microbial community to 3 days of fasting and further examine physiological effects caused by this feed deprivation.

## 2 MATERIALS AND METHODS

### 2.1 Ethics statement

All institutional and national guidelines for the care and use of laboratory animals were followed. The handling of the animals and the experimental procedures were approved by the Danish Animal Experiments Inspectorate.

### 2.2 Animals and housing

The 26 mink included in the study were 7‐month‐old males of the color type Brown (also referred to as color type Wild). Experienced animal caretakers on the research farm were responsible for the routine handling and care of the animals. The mink were housed individually in standard‐sized cages and were provided with a nesting box and bedding. The mink were fed twice a day (except during the fasting period) and had free access to tap water.

### 2.3 Experimental design

The study was designed as a parallel group experiment. The mink were divided into two groups at 3 months of age. The two groups were fed separate diets for 4 months: One group was fed a commercial mink diet from a feed kitchen (Feed_FC) and the other group was fed a diet locally produced at the research farm (Feed_West). Mink from two feeding groups on the research farm were included in order to evaluate if the feed composition would affect the microbiota of the mink.

The Feed_West was mainly based on fish by‐products (25%), boiled poultry offal (18%), cereals (15%), vegetable protein (6%), fat (11%), vitamins, and water; whereas, the Feed_FC contained 26% raw slaughter offal replacing some of the vegetable proteins and fat. The energy content was 9.1 versus 7.5 Mj/kg in Feed_west and Feed_FC, respectively, and the distribution of energy from protein, fat, and carbohydrates was 23:57:20 versus 28:56:16. At 7 months of age, 14 mink were subjected to 3 days of food deprivation (fasting) and subsequently killed. The remaining 12 mink were killed without a fasting period (nonfasting). The mink in the fasting and nonfasting groups included mink from both feed groups (Table 1). The fasting experiment was carried out from the 9th to the 11th of November 2014. Monitoring during the experiment included twice daily general health checks and a veterinary inspection by licensed veterinarians of all mink on the morning of the designated days of the fasting regimes. No morbidity or mortality occurred during the course of the experiment. All animals were killed on the same day, at the end of the 3‐day fasting period. Killing was performed by use of CO_2_ gas. The study was carried out at the Danish Fur breeders Association research farm, Kopenhagen Farm, Holstebro, Denmark.

**Table 1:** Experimental design and degree of fatty degeneration of hepatocytes

Experimental group	Fasted (n)		Nonfasted (n)	
Feed group	Feed_FC	Feed_West	Feed_FC	Feed_West
Experimental groups (n)	11	3	2	10
Severe fatty degeneration of hepatocytes^a^	8	1	0	0
Mild‐moderate fatty degeneration of hepatocytes^a^	3	0	0	0
<5% fatty degeneration of hepatocytes^a^	0	2	2	10
Total (n)	14		12	

a The evaluation of fatty degeneration were based on histopathological evaluation of liver biopsies and the classification based on a scoring system applied for classification of non‐alcoholic fatty liver syndrome (NAFLS).

### 2.4 Dissection of animals

Immediately after euthanasia, the stomach and intestines were removed from the animals as an entity for inspection and opening. A liver section from the left median lobe, pancreas, duodenum, and colon was placed in 10% neutral formalin to be processed for histology. After removing any fecal contents from the intestinal lumen, the mucosa layers were gently scraped off using sterile cell scrapers (VWR International, West Chester, PA). Mucosa scraps for microbiological culture and sequencing were placed in separate cryovials and frozen using dry ice. Scrap samples for microbiological culture were stabilized with 20% glycerol before freezing. The samples were kept on dry ice until they were transferred to storage at −80°C.

### 2.5 Bacterial culturing

Mucosal scraps collected from the colon from all 26 mink frozen in 20% glycerol as well as feed samples were used for plate culturing. Ten‐fold serial dilutions were prepared in peptone saline diluent and plated on Wilkins‐Chalgren agar (WCA, Oxoid), plate count agar (PCA, Oxoid), De man, Rogosa, Sharpe agar (MRS, Oxoid), and Bifidus selective agar (BSM, Sigma Aldrich) for enumeration of total anaerobic bacteria, total aerobic bacteria (and facultative anaerobic), lactobacilli, and bifidobacteria, respectively. Plates were incubated for 2‐3 days at 37°C anaerobically, except PCA plates were incubated aerobically.

### 2.6 Extraction of bacterial community DNA

Total community DNA was extracted from mucosal scrape samples collected from colon during dissection as well as from feed samples using the MoBio PowerLyzer^®^ Power Soil^®^ DNA Isolation Kit (MoBio Laboratories, Carlsbad, CA) according to the manufacturer's recommendations with minor modifications as previously reported (Bahl, Bergström, & Licht, 2012). DNA concentrations were measured with the Qubit dsDNA HS kit (Life Technologies).

### 2.7 Bacterial community composition and diversity

The bacterial community composition was determined by partial 16S rRNA gene sequencing of the extracted community DNA. Amplification of the V3‐region was performed following a dual PCR strategy. In the first amplification round, the universal bacterial primers PBU (5′‐CCTACGGGAGGCAGCAG‐3′) and PBR (5′‐ATTACCGCGGCTGCTGG‐3′) were used without sequencing adaptors. The PCR reactions were conducted with 2 μl AccuPrime PCR Buffer II, 0.5 μmol/L forward primer, 0.5 μmol/L reverse primer, 5 ng template DNA, and 0.08 μl AccuPrime Taq polymerase (Termo Fisher Scientific) in a total reaction volume of 20 μl. Reaction conditions were as follows: Initial 94°C for 2 min. followed by 30 cycles of 94°C for 20 s, 58°C for 20 s and 68°C for 30 s, and finally 68°C for 5 min. before cooling to 4°C. The PCR mix was subsequently diluted 10‐fold and 1 μl used as template in the second amplification step using the same forward primer with a unique 10–12 bp barcode for each bacterial community and reverse primer both with sequencing adaptors (Christensen et al., 2014). The PCR products (approximately 260 bp) were purified by gel electrophoresis and a library was constructed by combining equal amounts of PCR product from each sample. Sequencing was performed on a Ion Torrent PGM, using the Ion OneTouch^™^ 200 Template Kit v2 DL. Sequencing data were processed in CLC Genomics Workbench version 8.5 (Qiagen) for demultiplexing and removal of primers as previously described (Christensen et al., 2014). The output fasta files were analyzed using the pick_de_novo_otus.py pipeline with the usearch OTU picking method at 97% similarity and the RDP method with the Silva_111 database for taxonomic assignment (0.5 confidence) incorporated in the QIIME pipeline (Caporaso et al., 2010). Filtering of OTU's was applied to include only OTU's classified as bacteria and removal of singletons and those present in only one sample. Alpha diversity (OTU richness and Shannon diversity index) and beta diversity (principle coordinate‐based analysis based on unweighted UniFrac distance matrix) were calculated within the Qiime pipeline. The final number of reads per sample ranged from 4,592 to 54,852 (average 26,821) in the 15 mink samples (eight fasted and seven nonfasted) and two feed samples (in triplicates) included in the analyses. Sequencing data are deposited at NCBI Sequence Read Archive with the accession number SRP091648.

### 2.8 Data handling and statistics

Statistical analysis was conducted in GraphPad Prism (version 5.03; GraphPad Software Inc., La Jolla, CA) unless otherwise stated. Differences between the fasted and nonfasted groups were assessed by unpaired t‐tests or nonparametric Mann‐Whitney tests as appropriate. Identification of differentially abundant bacterial taxa based on 16S rRNA gene sequencing was performed by permutation tests adjusted for multiple comparisons with a false discovery rate threshold q = 0.05 (Pike, 2011).

## 3 RESULTS

### 3.1 Pathology and histopathology

Macroscopical evaluation revealed mild to moderate fatty degeneration in the liver of all fasted animals. The liver sizes were normal to slightly increased and the liver tissues were light in color and friable of texture. Other organs appeared normal. Results of histopathological evaluation of liver biopsies are presented in Table 1. Severe fatty degeneration of hepatocytes was recorded in 64% (9/14) of the fasted animals and in none of the nonfasted animals. Moderate to mild fatty degeneration of hepatocytes were recorded in 21% (3/14) of the fasted animals and in none of the nonfasted animals. There were no recorded histopathological lesions in biopsies from intestines and pancreas.

### 3.2 Bacterial culturing

**Figure 1:**
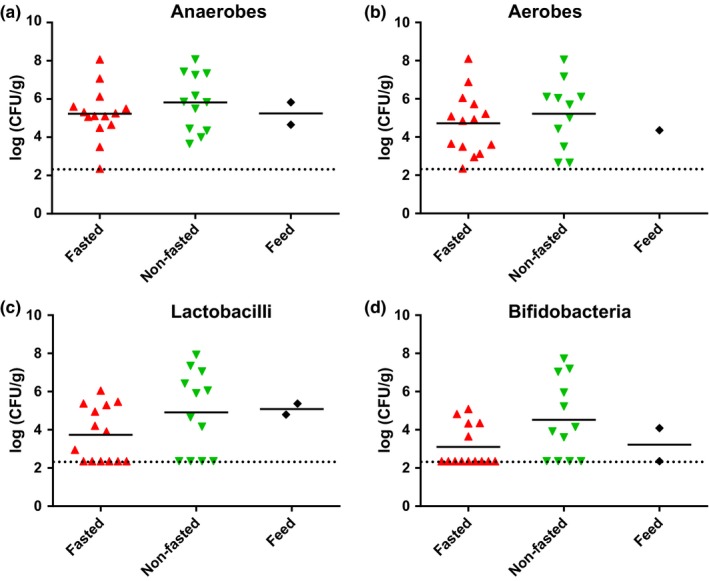
Effects of fasting on the culturable bacterial load in mucosal scraps. (a) Total anaerobic bacteria (Wilkins‐Chalgren agar), (b) total aerobic bacteria (plate count agar), (c) lactobacilli (de Man Rogosa agar), and (d) bifidobacteria (BSM agar) in mucosal scraps from fasted and nonfasted animals as well as feed samples. In all panels, counts from individual animals are shown with lines representing geometric averages. No significant differences between groups were found

The average number of colony‐forming units in mucosal scrape samples taken from fasted and nonfasted animals determined on four different agar media showed no difference between the two groups (Figure 1). The range of both aerobic and anaerobic cultivable bacteria in individual samples was found to be from below the detection level (225 CFU/g) to approximately 10^8^ CFU/g indicating a large variation between animals. The average levels of both lactobacilli and bifidobacteria appeared lower than the total anaerobic counts and were undetected in many animals, however, in some animals, both bacterial groups reach levels of 10^8^ CFU/g.

### 3.3 Bacterial community diversity and composition

**Figure 2:**
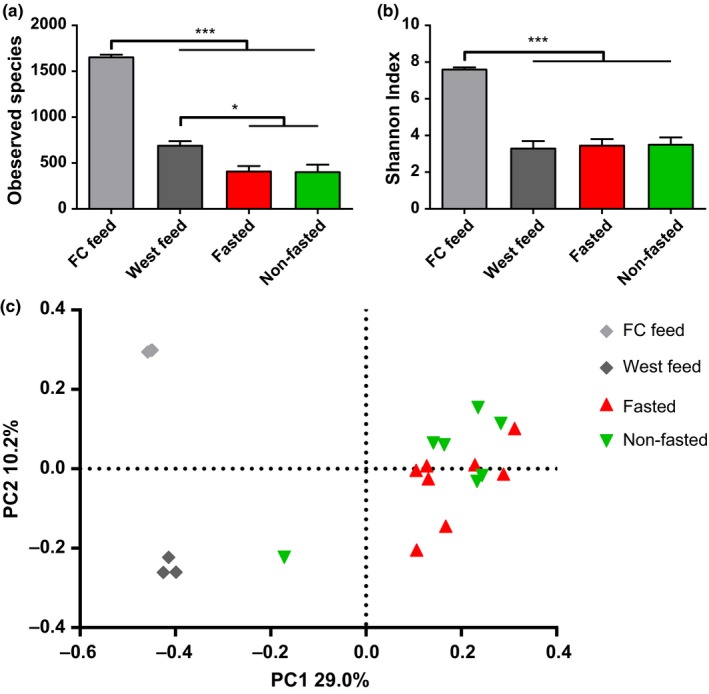
Alpha‐ and beta diversity of the bacterial communities in the mucosal scraps of fasted and nonfasted animals. (a) Bacterial richness (Number of observed OTU's) and (b) Shannon diversity index from mucosal scraps from fasted and nonfasted animal as well as feed samples. Columns show mean values with SEM indicated by error bars. Significant differences between groups are indicated by asterisks (*p < .05, ***p < .001). (c) Beta diversity represented as principle coordinate analysis based on unweighted UniFrac matrix. Each dot represents one mucosal scrap from fasted or nonfasted animals as well as feed samples

**Figure 3:**
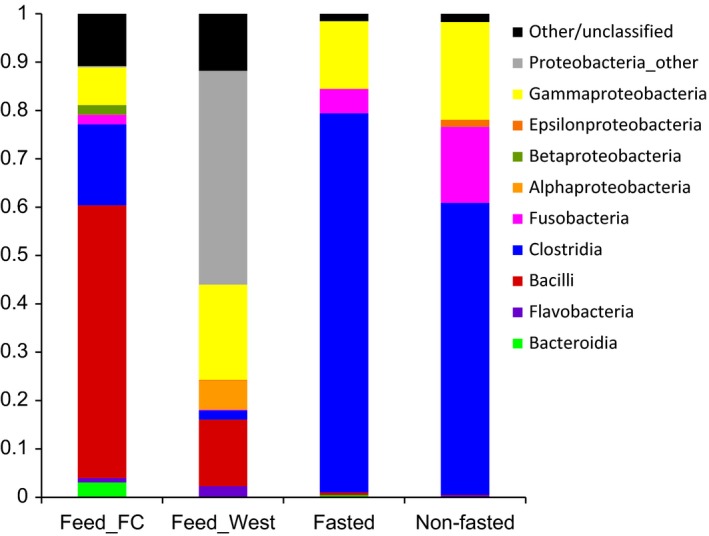
Bacterial community composition based on 16S rRNA gene sequencing. The average bacterial composition at the class level for each feed type (Feed_FC and Feed_West) and mucosal scrap samples from the fasted and nonfasted animals are shown

**Figure 4:**
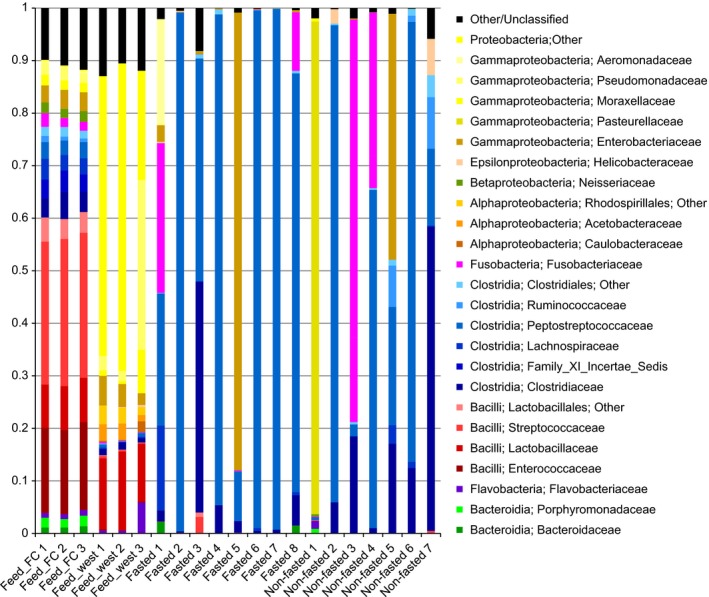
Bacterial community composition of individual animals and feed samples. The bacterial composition at the family level is shown for each mucosal scrap sample obtained from individual animals as well as the triplicate feed samples (Feed_FC and Feed_West)

No differences were found in estimations of alpha diversity (OTU richness and Shannon diversity index) and beta diversity between the fasted and nonfasted animals (Figure 2a–b). There was, however, a significantly higher richness in both types of feed compared to the richness found in the mucus samples and the Shannon diversity index was also higher for the Feed kitchen (Feed_FC) diet (Figure 2b). Beta diversity analysis showed that samples obtained from fasted and nonfasted animals clustered together and separately from the feed samples, which formed two distinct clusters (Figure 2c). At the class level, the average bacterial composition in both the fasted and nonfasted group was dominated by Clostridia, Gammaproteobacteria, and Fusobacteria, which appears markedly different from both feed types (Figure 3). A total of 18 bacterial families were found to represent at least 1% of the total community in an animal across all mucosal samples. No differences in the relative abundance of any of these families were found between the fasted and nonfasted animal, however, a high degree of bacterial community variation in individual animals within and between the groups was observed (Figure 4).

## 4 DISCUSSION

Due to the short intestinal tract and fast transit time in the gut of the mink, one could speculate that the microbial community of mink may simply reflect that of their feed. It has been suggested, that the rapid movement of food through the gastrointestinal tract may not allow enough time for bacterial metabolism to provide an environment that is suitable for growth of anaerobes (Williams et al., 1998). In order to investigate this, we examined the mucosa‐associated bacterial composition in the mink gut under normal feeding conditions and after a 3‐day fasting period. We reasoned that the bacterial community present following a 3‐day fasting period would represent the colonizing and established microbiota of the animals. As expected, we found large differences between the bacterial community composition of the two feed types and the animal samples. However, we found no significant differences in the mucosa‐associated microbiota between fasted and nonfasted animals irrespective of feed type (Figure 3), although some individual animals had markedly different microbial community composition (Figure 4). This suggests that the mucosa‐associated microbiota is largely unaffected by 3 days of fasting in the examined farmed mink. This is further supported by the culture data, which revealed no differences in the mean number of live mucosa‐associated bacteria between the fasted and nonfasted animals. A large variation between individual animals in the number of CFUs on the different culture media was found (Figure 1), which is consistent with the differences found in sequencing data and previous culture‐based studies (Pedersen & Jorgensen, 1992; Williams et al., 1998). Further studies are necessary to assess whether the large interindividual differences in bacterial composition and number of CFUs may play a role in health and disease of the animals.

Although most animals were found to be dominated by bacteria belonging to the Firmicutes class Clostridia as also reported in fecal samples from other carnivorous animals (Garcia‐Mazcorro et al., 2012; Gugołek et al., 2015; Handl et al., 2011), it is interesting to observe that the mucosa‐associated microbiota of some animals was vastly dominated by other bacterial groups including *Fusobacteriaceae*,* Pasteurellaceae*, and *Enterobacteriaceae* (Figure 4). Species within these bacterial families are associated with pathogenicity (Hunter, 1996; Löliger, 1970), although no adverse effects were observed in this study. Further studies are needed to address the association between health issues and gut microbiota in farmed mink.

The bacterial culturing data show comparable levels of anaerobic and aerobic bacteria with an average level of approximately 10^5^–10^6^ CFU/g, which may, in part, be due to difficulties culturing many of the clostridia identified by sequencing, and is consistent with a previous study enumerating the culturable bacteria from the small intestine mucosa (Williams et al., 1998). Interestingly, Williams et al. also found no effect on colonization level of the mucosa after 36 hr of food deprivation, supporting the idea that mucosa‐associated bacteria represent a more permanent community than that found in luminal content. Counts of both lactobacilli and bifidobacteria levels varied from below detection level to levels comparable to the total anaerobic cell count in some animals. This is consistent with a previous study in ferrets, which showed that these two genera to be the second and third most frequently isolated anaerobic bacteria (Nizza et al., 2014), but seems inconsistent with the 16S rRNA gene sequencing data, which indicate very low relative abundance of lactobacilli and no bifidobacteria. Reasons for this discrepancy may include the relatively high detection level of the sequencing‐based approach compared to culturing and the inability to culture all anaerobic bacteria from the samples with the method used (Rajilić‐Stojanović & de Vos, 2014).

Fasting expectedly caused slight weight loss. Fasting is known to result in the development of fatty liver in carnivores (Mustonen et al., 2005; Nieminen et al., 2009; Rouvinen‐Watt et al., 2010; Szabo et al., 2000). All fasted mink in the study developed varying degrees of fatty degeneration of hepatocytes (Table 1), though none of the mink developed severe fatty liver syndrome. It has been reported that mink tolerate fasting for 3 days, while longer fasting periods induce development of severe fatty liver syndrome (Rouvinen‐Watt et al., 2010) and the risk of development of severe fatty liver syndrome may be elevated in some mink with excess body fat due to high feeding intensity (Dick et al., 2014). The normal body condition of the mink in this study and a fasting period limited to 3 days, would have decreased the risk of lipid degeneration progressing into severe fatty liver. It has been reported that fasting in mink may induce decreased intestinal mass as well as both histomorphometric and biomechanical remodeling of the intestinal tract (Bjornvad, Elnif, & Sangild, 2004). The intestinal tract of both fasted and nonfasted animals appeared normal at gross and histopathological evaluation. It was not within the scope of this study to do a complete morphometric and biomechanical assessment of the biopsies from the intestine, and therefore, some remodeling changes may have remained undetected by the histopathological evaluation. Since there was no detected difference in bacterial diversity and composition between fasted and nonfasted animals, the mucosa‐associated mink gut microbiota seemed to remain largely unaffected by the 3‐day food deprivation as well as any physiological changes induced by this. It should, however, be noted that large variations in bacterial composition was observed between individual animals, which to some extent is expected to mask the effects of fasting, due to the low number of animals included in the study.

To the best of our knowledge, this is the first study to apply high‐throughput 16S rRNA gene sequencing to characterize the gastrointestinal microbiota in the mink. This study only included a limited number of apparently healthy mink, but the methodology is of substantial interest to yield new insights into the pathogenesis of many diseases in the mink production as well as defining new prophylactic and therapeutic interventions.

## CONFLICT OF INTEREST

The authors declare no financial or personal conflicts of interest.
